# Novel *USP18* mutations lead to severe interferonopathy responsive to JAK inhibitor

**DOI:** 10.3389/fimmu.2025.1646996

**Published:** 2025-09-04

**Authors:** Xiangwei Sun, Ming Li, Yiying Dou, Qintao Wang, Jianming Lai, Jia Zhu, Qing Zhou, Xiaomin Yu

**Affiliations:** ^1^ Liangzhu Laboratory, Zhejiang University, Hangzhou, China; ^2^ Department of Rheumatology, Sir Run Run Shaw Hospital, Zhejiang University School of Medicine, Hangzhou, China; ^3^ Department of Rheumatology and Immunology, Capital Center for Children’s Health, Capital Medical University, Beijing, China

**Keywords:** USP18, type I interferonopathies, autoinflammation, type I interferon, targeted therapy

## Abstract

**Introduction:**

Ubiquitin-specific peptidase 18 (USP18) is a key negative regulator of type I interferon (IFN) signaling. USP18 deficiency resulted in embryonic or neonatal lethality with severe systemic inflammation and neurological anomalies due to excessive IFN signatures. Importantly, additional disease-causing USP18 mutations remain to be identified and functionally characterized.

**Methods:**

Whole-exome sequencing was performed to identify pathogenic variants in two affected individuals. Extensive immunologic and functional assay were used to characterize inflammatory signatures and evaluate the impact of the variants on type I IFN signaling. Therapeutic intervention with the JAK inhibitor was administered and clinical response was monitored.

**Results:**

We identified novel USP18 biallelic mutations (p.C230X and p.G317S) in siblings with severe early-onset systemic inflammation. Patient PBMCs exhibited hypersensitivity to IFNα, leading to aberrant and prolonged activation of type I IFN signaling. Mechanistic studies revealed that the p.G317S variant disrupted the interaction between USP18 and ISG15, thereby impairing its negative regulatory function. Treatment with JAK inhibitor ruxolitinib alleviated the inflammatory phenotypes, followed by a sustained recovery.

**Conclusion:**

Novel biallelic mutations of USP18 lead to excessive type I IFN responses and severe interferonopathy. Our findings highlight a novel pathogenic mechanism in which impaired ISG15 binding compromises the regulatory function of USP18. The favorable clinical response to ruxolitinib suggests a promising therapeutic strategy.

## 1 Introduction

Type I interferons (IFNs), primarily IFNα and IFNβ, are a group of cytokines with antiviral, immune regulatory, and anti-proliferative functions. However, dysregulated type I IFN signaling has been implicated in the onset of autoinflammatory and autoimmune diseases (1, 2). Type I interferonopathies are monogenic disorders characterized by the overproduction of, or heightened responsiveness to type I IFNs, leading to aberrant activation of the type I IFN signaling pathway. Clinically, these disorders present with a broad spectrum of phenotypes, ranging from early lethality and severe neurological manifestations to mild cutaneous symptoms, with brain, lung and skin being the most commonly affected organs. The molecular mechanisms involve increased sensitivity or constitutive activation of nucleic acid sensors, loss of protective nuclease activity, failure of proteasome complex formation, and disruption of the negative regulation of the type I IFN signaling pathway (3). Currently, more than 35 genes have been identified as causative for type I interferonopathies (4).

Ubiquitin-specific peptidase 18 (USP18) is an IFN-inducible protein and serves as a key negative regulator of type I IFN signaling (5). USP18 specifically binds to the intracellular domain of the type I interferon receptor 2 (IFNAR2), interfering with the JAK1-receptor interaction and thereby preventing the downstream phosphorylation cascade and the expression of IFN-stimulated genes (ISGs). The interaction between USP18 and IFNAR2 relies on cooperative transport and interactions of USP18 and STAT2 (6). In addition, USP18 is an interferon-stimulated gene 15 (ISG15)-specific isopeptidase that removes the ubiquitin-like protein from ISGylated proteins (deISGylation). Its negative regulatory role in type I IFN signaling is independent of its enzymatic activity (7). Synergistically, ISG15 stabilizes USP18 against proteasomal degradation, regardless of their affinity (8). USP18 deficiency causes embryonic or neonatal lethality with severe systemic inflammation and neurological anomalies due to excessive IFN signatures (9–11). Three documented cases of *USP18* loss-of-function (LOF) mutations exhibit distinct pathogenic mechanisms: complete absence of USP18 protein, defective stabilization by ISG15, and impaired STAT2 binding (9–11). Given the critical role of USP18, the pathogenicity of additional *USP18* mutations, as well as the mechanisms driving exaggerated IFN response, requires further investigation.

In this study, we identified two siblings with novel mutations in *USP18* gene who presented with severe early-onset autoinflammatory disease and elevated IFN signatures. The clinical phenotypes and heightened sensitivity to type I IFN stimulation were consistent with previously reported USP18 deficiency. We propose a novel pathogenic mechanism that has not yet been investigated *in vivo*. Our findings expand the understanding of USP18-related pathogenesis and promote the use of targeted therapeutic drugs.

## 2 Materials and methods

### 2.1 Patients

Patients enrolled in the study were evaluated under a protocol approved by Children’s Hospital, Zhejiang University School of Medicine (2021-IRB-172). Written informed consent was provided by the parents of the patients.

### 2.2 Whole exome sequencing and Sanger sequencing

Whole exome sequencing (WES) analysis was performed on genomic DNA isolated from peripheral blood from patients and their parents. WES data was analyzed as previously described (12, 13). Briefly, multiple in-silico tools, including *BWA*, *Samtools*, *GATK 4.0* and *ANNOVAR*, were employed. The human reference genome (GRCh38) was used for alignment and variant annotation. Then based on factors such as allele frequency, predicted pathogenicity and inheritance pattern, candidate genes were filtered and confirmed by Sanger sequencing.

### 2.3 Bulk RNA sequencing

Total RNA was extracted from PBMCs using the RNeasy Mini Kit (Qiagen, 74104). The quality and purity of RNA were assessed by an Agilent Bioanalyzer RNA chip. Next, RNA was reverse-transcribed into cDNA, followed by fragmentation into 150 – 200 base pair paired-end reads, RNA libraries were then generated using NEB Next Ultra RNA Library Prep Kit for Illumina (New England Biolabs). Sequencing was performed on Illumina NovaSeq. The quality-controlled reads were aligned to GRCh38 using *HISAT2*, and read counts mapped to each gene were determined with *featureCounts*. Differential expression analysis was conducted with the *DESeq2* R package, and downstream heatmap visualization was performed using the R package *pheatmap*.

### 2.4 Calculation of interferon score

The 28 genes associated with type I interferon signaling were selected as previously described (14). Z-scores for each of these genes were calculated from *DESeq2*-normalized RNA-seq counts, using the mean and standard deviation of healthy control samples under unstimulated condition as the reference. At last, the interferon score for each sample was then determined as the sum of the Z-scores for all 28 genes.

### 2.5 Quantitative RNA

RNA extraction was as described above. Next, 1 μg RNA was reverse transcribed into cDNA with Vazyme HiScript IV All-in-One Ultra RT SuperMix for qPCR (R433). qPCR was performed using ChamQ Blue Universal SYBR qPCR Master Mix (Q312 - 02) on Roche’s LightCycler480 qPCR system. Relative mRNA expression levels were normalized to housekeeping gene GAPDH and analyzed by the ΔΔCt method.

### 2.6 Cell preparation, culture, and stimulation

PBMCs were separated from peripheral blood using lymphocyte separation medium (MP, 50494). HEK293T and THP - 1 cell lines were obtained from the American Type Culture Collection. PBMCs and THP - 1 cells were cultured in RPMI 1640 (Gibco, C11875500BT) supplemented with 10% fetal bovine serum (Vazyme, F103) and penicillin/streptomycin (Thermo Fisher Scientific, 15140163). HEK293T cells were grown in Dulbecco’s Modified Eagle Medium (Gibco, C11995500BT) supplemented with 10% FBS and 1% penicillin/streptomycin.

THP-1 *USP18* KO monoclonal cell lines were generated by CRISPR-Cas9 system targeting sequence (TCAGGTGTTCGTAATGAATG) of exon 2 of *USP18*, followed by selection with 1ug/ml puromycin. Gene deletion was confirmed by Sanger sequencing and western blotting. Subsequently, monoclonal cells were stably transduced with lentivirus carrying WT or mutant USP18 cDNA.

IFNα (Miltenyi Biotec) was used to stimulate PBMCs, HEK293T cells and THP - 1 cells for the indicated amount of time and concentrations.

### 2.7 Expression plasmids and antibodies

Human USP18, IFNAR2, STAT2, ISG15, HERC5, UBA7, and UBCH8 cDNA were amplified from PBMCs and then cloned into vectors with different tags in the C-terminal or N-terminal end to construct plasmids. Mutant USP18 plasmids were generated by site-directed mutagenesis. Plasmids used in this study included pcDNA3.1-USP18-Flag, pCMV-USP18-HA, lenti-myc-ISG15, lenti-BSD-USP18-Myc-Flag, lenti-STAT2, lenti-Flag-STAT2, pcDNA3.1-IFNAR2-ICD-Flag, pXN-myc-His-UBA7, pXN-myc-His-UBCH8 and pcDNA3.1-Herc5-HA.

Western blotting and flow cytometry were performed using a variety of antibodies: STAT1 (Cell signaling technology, 14994), Phospho-STAT1 (Cell signaling technology, 9167), STAT2 (Cell signaling technology, 72604), Phospho-STAT2 (Cell signaling technology, 88410), HA (Cell signaling technology, 3724), USP18 (Cell signaling technology, 4813), ISG15 (Santa Cruz, 166755), FLAG (HUABIO, 0912 - 3), HRP-conjugated GAPDH (Proteintech, 60004), CD3-APC-H7 (BD Biosciences, 560176), CD14-PE-CY7 (BD Biosciences, 557742), CD19-BB700 (BD Biosciences, 566396) and BD Phosflow™ PE Anti-Human Stat1 (pY701) (BD Biosciences, 612564).

### 2.8 Western blotting and co-immunoprecipitation

Cells were harvested and lysed in an appropriate volume of cold 0.5% NP40 lysis buffer freshly supplemented with protease and phosphatase inhibitors (Thermo Fisher Scientific, 78442) for 30 minutes, centrifuged at 13,000 × rpm, 4°C for 15 min. Protein concentration was determined by Pierce™ BCA Protein Assay Kits (Thermo Fisher, 23225).

For co-immunoprecipitation, 5% of the collected cell lysate was used for “input” and the remaining sample was incubated with magnetic beads overnight at 4 °C in a revolving rack. After incubation, the beads were washed with wash buffer three times and protein complex was directly eluted by boiling in 1 x SDS loading buffer at 95 °C for 5 minutes, then separated through routine SDS-PAGE and transferred to 0.45 μm nitrocellulose membrane (Cytiva, 10600002) and blotted with indicated antibodies.

### 2.9 Flow cytometry analysis

For phosflow staining, PBMCs was plated at a density of 1×10^^6^ cells/mL and stimulated with IFNα (1ng/mL) for 30 minutes at 37 °C, with 5% CO_2_. After stimulation, surface antibodies were added and stained at room temperature for 30 minutes after removing the stimulation. Following surface staining, cells were fixed with paraformaldehyde (PFA) and permeabilized with 40% methanal, and stained by phosphorylation-specific antibodies to assess the activation levels of inflammatory signaling pathways. Data were acquired on a CytoFlex S flow cytometer (Beckman Coulter) and analyzed with FlowJo software (Tree Star).

### 2.10 Dual-luciferase reporter assay

HEK293T cells were seeded in a 24-well plate at a density of 2×10^^5^ cells per well and transiently transfected with ISRE-dependent Dual Luciferase Reporter plasmids, along with WT or G317S mutated USP18 plasmids. At 24 hours post-transfection, cells were stimulated with indicated concentration of IFNα for 6 hours and luciferase activity was measured using luciferase assay (Vazyme, DL101 - 01) on a multimode plate reader (BioTek). Data was analyzed as fold induction by normalizing Firefly luciferase activity to Renilla luciferase activity.

### 2.11 Statistical analysis

Graphing imaging were performed using *R* version 3.5.2 and *PyMOL* version 3.0.3. Statistical analysis was performed using *GraphPad Prism 8* software (GraphPad Software). Data for statistical tests were derived from three or more independent biological replicates and all values were expressed as mean ± SD. For comparisons between two groups, the two-tailed unpaired t-test was applied for significance analysis; for comparisons involving more than two groups, analysis of variance (ANOVA) was employed. In all tests, a 95% confidence interval was used, for which P < 0.05 was considered a significant difference.

## 3 Results

### 3.1 Biallelic *USP18* mutations identified in patients with severe inflammatory disease

**Figure 1:**
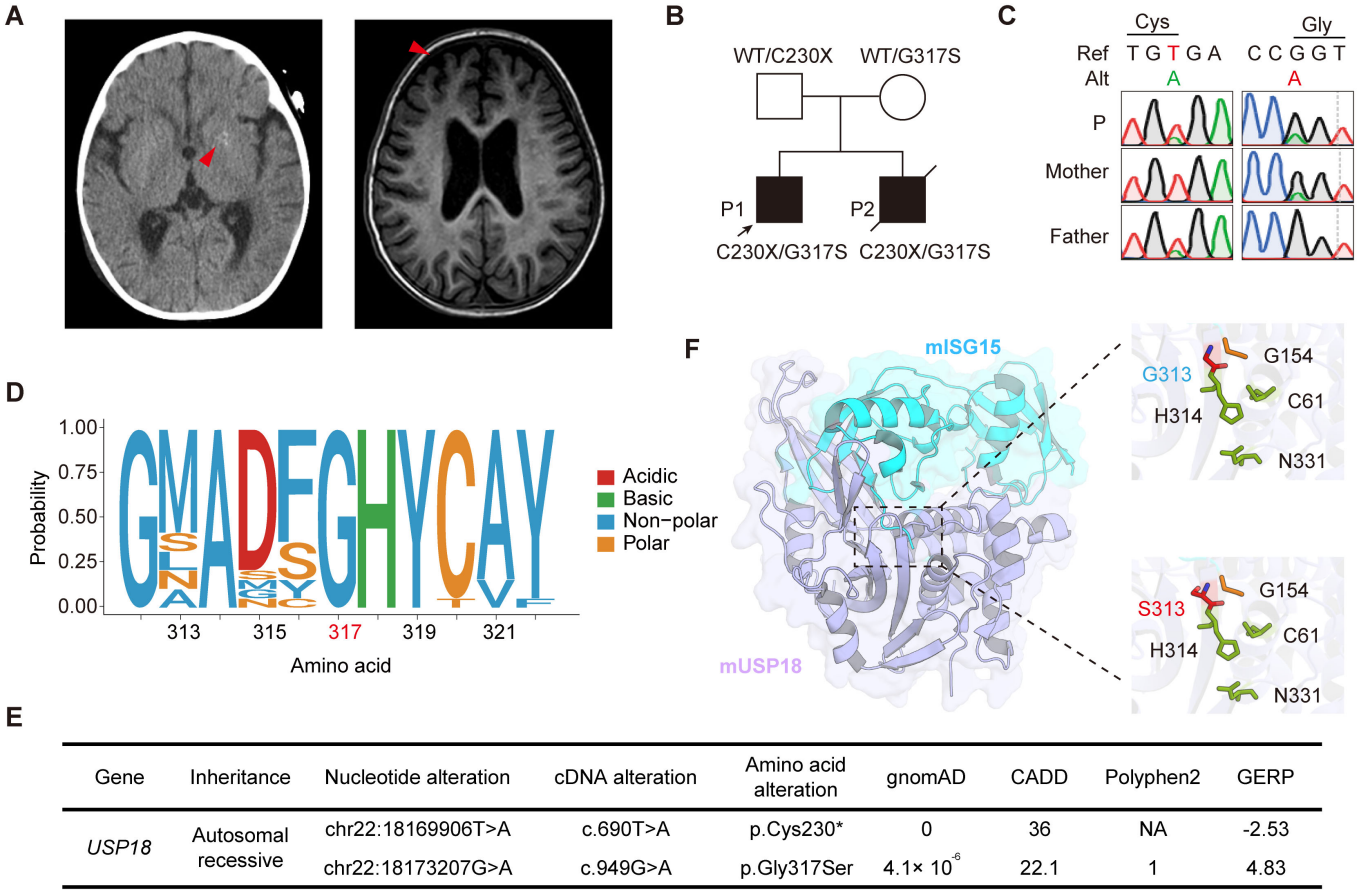
Identification of compound heterozygous *USP18* C230X/G317S mutations in patients with severe early-onset systemic inflammation. **(A)** Radiological manifestations of patient P1. Intracranial calcification imaged by CT and cerebral sulcal widening imaged by MRI (red arrows). **(B)** Pedigrees of the patients with compound heterozygous C230X/G317S mutations in *USP18*. **(C)** Validation of the *USP18* mutations of family by Sanger sequencing. **(D)** The evolutionary conservation of the USP18 G317 site across species. **(E)** Frequency and pathogenicity prediction of *USP18* mutations. **(F)** Crystal structure of mUSP18 (purple) complexed with mISG15 (blue) (PDB:5CHV). Expanded views show the mISG15 C-terminal tail engaging with the catalytic triad of mUSP18 (C61, H314, and N331, green). G313 (blue) or S313 (red) in mUSP18 was above the catalytic triad. G154 (yellow) was located in the C-terminal of mISG15. The figure was performed using *PyMOL*.

Patient 1 (P1) is a two-year-old male with a history of recurrent respiratory tract infections since the age of 6 months. At one year, he was hospitalized for acute pneumonia, fever, and respiratory distress. Additionally, he presented with systemic inflammation and multiorgan dysfunction, including leukopenia, thrombocytopenia, severe anemia, hypoalbuminemia, hepatosplenomegaly, acute cardiac insufficiency, and acute kidney injury. Computed tomography (CT) neuroimaging demonstrated multifocal punctate calcifications in the bilateral cerebellar hemispheres and basal ganglia nuclei, while magnetic resonance imaging (MRI) revealed prominent cerebral sulcal widening in the frontotemporal regions, indicative of neuroinflammatory conditions (**Figure 1A**). Laboratory investigations showed significantly elevated levels of C-reactive protein (CRP), erythrocyte sedimentation rate (ESR), and ferritin. Microbiological studies identified co-infection with influenza B virus and methicillin-sensitive Staphylococcus aureus. No autoimmune autoantibodies were detected in patient P1 (**Supplementary Table S1**).

Patient 2 (P2), the patient P1’s brother, had a fatal clinical course, characterized by acute hemorrhagic pneumonia at three months of age, complicated by hypoalbuminemia, disseminated intravascular coagulation, refractory cerebral edema, and seizure activity. Despite intensive care support, the patient succumbed to the condition on the 11th day of hospitalization (**Supplementary Table S2**).

Whole exome sequencing of the family was performed, and we identified that patients P1 and P2 carried compound heterozygous mutations in the *USP18*: NM_017414: c.690T>A and c.949G>A, resulting in an early stop-gain in Cys230 (C230X) and a Gly317Ser (G317S) substitution, respectively. Their healthy parents were heterozygous carriers, consistent with segregation of an autosomal recessive inheritance pattern (**Figure 1B**). The mutations were also confirmed by Sanger sequencing (**Figure 1C**). The G317S variant was highly evolutionarily conserved across different species (**Figure 1D**). The C230X variant was absent from all publicly available databases of genomic variation and the G317S variant was found in the heterozygous state at an extremely low frequency of 4.1 × 10^^-6^ in the gnomAD 2.1.1 database. Both of them were predicted to be deleterious by multiple silico tools (**Figure 1E**). USP18 possesses a three-domain architecture characteristic of cysteine proteases with a typical catalytic triad, which enables it to specifically remove ubiquitin-like modifier ISG15 from its conjugated proteins. The crystal structure of murine USP18 (mUSP18), both alone and in complex with mISG15, has been extensively studied (15). The G317 residue in human USP18 (hUSP18) corresponds to G313 in mUSP18. In close-up views of the mISG15-bound mUSP18, G313 is positioned above the typical catalytic triad (C61, H314, and N331), with its side chain oriented toward the C-terminal tail of mISG15 (G154) (**Figure 1F**). Collectively, the familial segregation, rarity of the allele, absence of other candidate genes, and the potentially damaging impact of the substitutions suggested that both variants could be disease-causing variants.

### 3.2 Activation of type I IFN signaling in the patient

**Figure 2:**
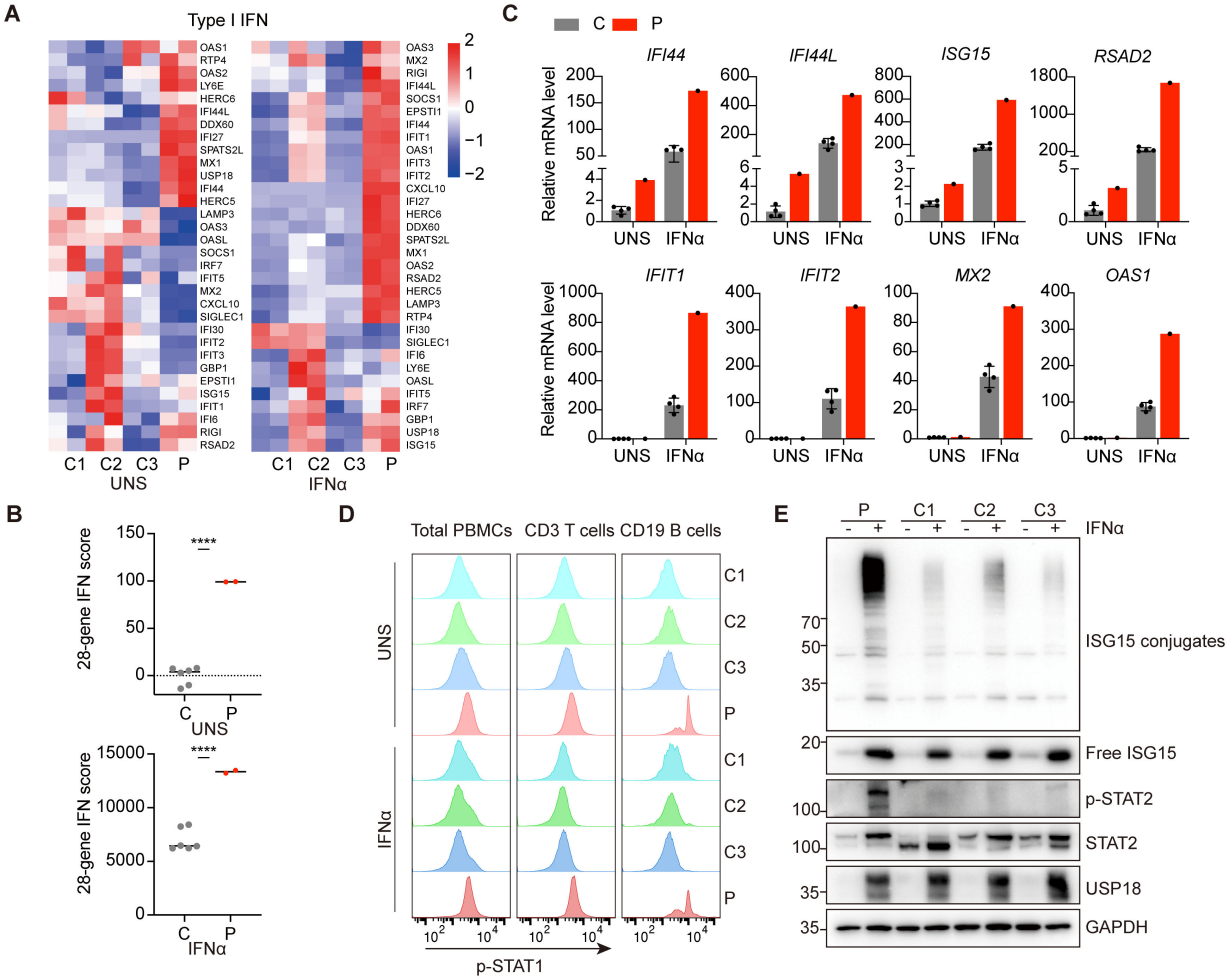
Hyperactivation of type I IFN signaling and heightened sensitivity to IFNα of patient PBMCs. **(A)** RNA-sequencing analysis of genes involved in the type I IFN pathway in PBMCs from patient P1 (P) and three healthy controls (C1-C3) under untreated or 10 ng/ml IFNα stimulation for 6 hours. Analysis of each sample was performed in duplicate. **(B)** Quantification of 28-gene IFN score of RNA sequencing data from **(A)**. All scores were normalized to healthy controls **(C)** under unstimulated conditions (UNS). Data are mean ± SD. *P* values determined by unpaired two-tailed t-test. ****P < 0.0001. **(C)** qPCR analysis of transcriptional levels of ISGs in PBMCs from patient P1 (P) and healthy controls **(C)** under untreated or 10 ng/ml IFNα stimulation for 6 hours. **(D)** Flow cytometry analysis of phosphorylation levels of STAT1 in patient P1 (P) and healthy controls (C1-C3) total PBMCs, CD3^+^ T lymphocytes and CD19^+^ B lymphocytes under untreated or 10 ng/ml IFNα stimulation for 30 minutes. **(E)** Western blotting analysis of PBMCs from patient P1 (P) and three healthy controls (C1-C3) with or without IFNα treatment for 24 hours by using the indicated antibodies.

The clinical phenotypes of the patients were reminiscent of a particularly severe form of type I interferonopathies. Consequently, we investigated the inflammatory signatures, focusing on type I IFN signaling in patient P1. RNA sequencing implicated elevated transcription of genes involved in type I IFN pathway in patient P1 peripheral blood mononuclear cells (PBMCs) compared to healthy controls (**Figure 2A**). Since the interferon gene expression scores can serve as potential diagnostic biomarkers for IFN-mediated diseases, we calculated a 28-gene IFN score based on type I IFN gene sets. The results confirmed the activation of type I IFN signaling in the patient (**Figure 2B**). Quantitative PCR analysis further validated the transcriptional upregulation of ISGs in patient P1 PBMCs, such as *IFI44*, *IFI44L*, *ISG15* and *RSAD2* (**Figure 2C**). Flow cytometry analysis revealed that the level of phosphorylated STAT1 (p-STAT1) was strikingly enhanced in CD19^+^ B lymphocytes, with a milder increase in total PBMCs and CD3^+^ T lymphocytes from patient P1 compared to healthy controls under unstimulated conditions (**Figure 2D**). USP18 is strongly induced by type I IFN treatment and is essential for suppressing persistent activation of the pathway. Cells derived from patients with USP18 deficiency exhibited heightened sensitivity to type I IFNs stimulation (9–11). Accordingly, we treated PBMCs from patient P1 and healthy controls with IFNα. RNA sequencing and quantitative PCR analysis revealed elevated transcriptional levels of ISGs in patient P1 PBMCs (**Figures 2A-C**). p-STAT1 level was also robustly upregulated, confirmed by flow cytometry analysis (**Figure 2D**). Immunoblotting further showed increased global ISGylated protein levels and persistent phosphorylated STAT2 in patient PBMCs upon IFNα stimulation compared to healthy controls (**Figure 2E**). Additionally, inflammatory cytokines, IL - 6, IL - 1β, TNF-α, and chemokine IL - 8 were prominently elevated in the patient P1 serum (**Supplementary Table S1**). Collectively, these findings indicated patient P1 exhibited activated inflammatory responses, particularly in the type I IFN pathway, along with an enhanced response to IFNα.

### 3.3 The *USP18* G317S variant is a loss-of-function variant that disrupts interaction with ISG15

**Figure 3:**
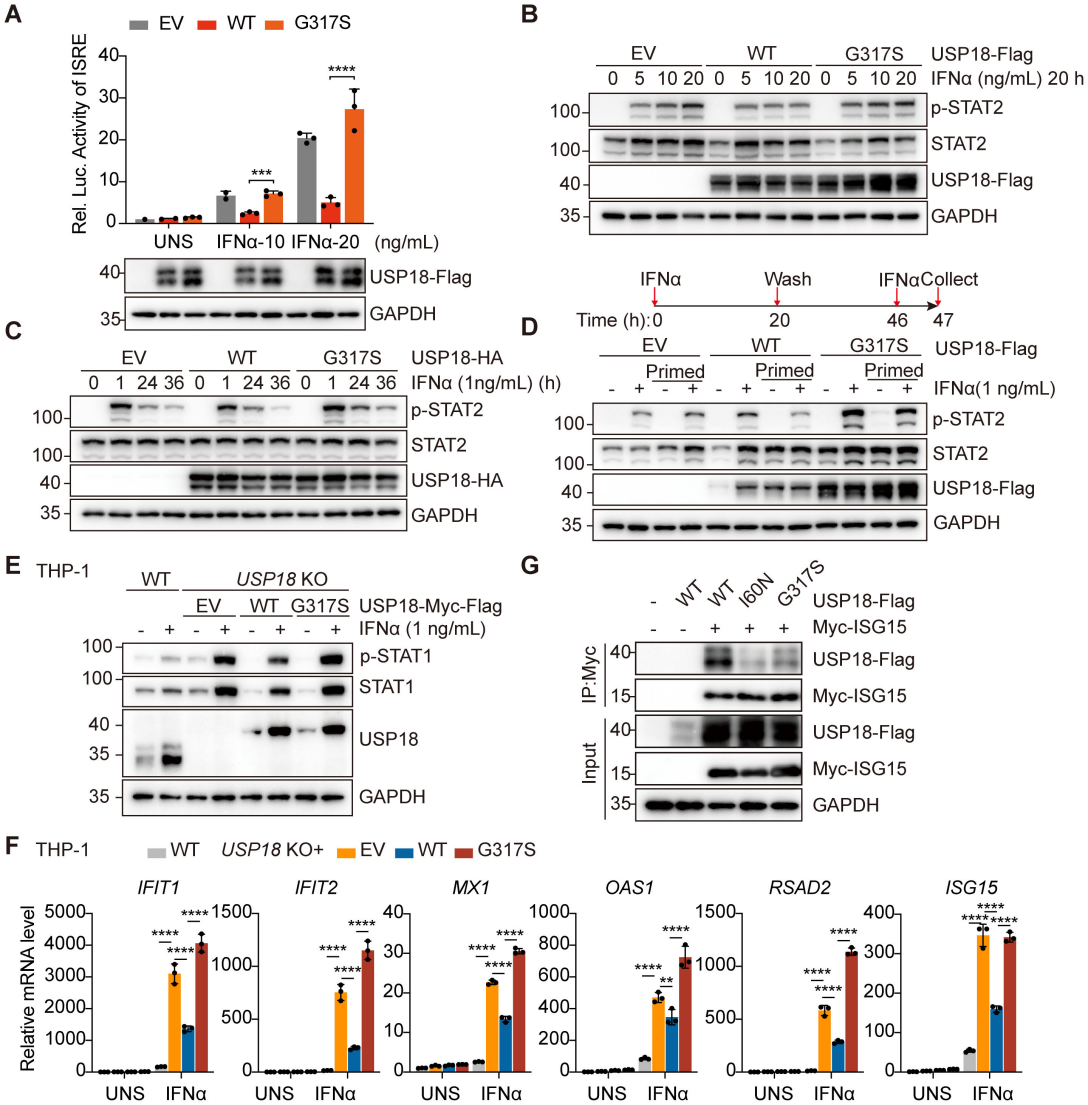
The G317S mutant USP18 compromised negative regulation of type I IFN signaling through its impaired interaction with ISG15. **(A)** ISRE activity determined by luciferase report gene assay in HEK293T cells overexpressing empty vector (EV), wild-type (WT) or G317S mutant USP18 upon indicated IFNα stimulation. Data are mean ± SD. *P* values determined by unpaired two-tailed t-test. ***P < 0.001, ****P < 0.0001. **(B, C)** Western blotting of the activation of type I IFN signaling indicated by p-STAT2 levels in transiently transfected HEK293T cells treated with IFNα stimulation at increasing concentrations **(B)**, for varying time points **(C)**. EV, empty vector; WT, wild-type USP18; G317S, G317S mutant USP18. **(D)** Western blotting of levels of p-STAT2 induced by IFNα in unprimed and primed cells. Cells were left untreated or stimulated (prime) with IFNα for 20 hours, then extensively washed and allowed them rest for 26 hours before being restimulated with IFNα for 1 hour. EV, empty vector; WT, wild-type USP18; G317S, G317S mutant USP18. **(E)** Western blotting of the activation of type I IFN signaling indicated by p-STAT1 levels in THP - 1 wild-type (WT) and *USP18* KO cells reconstituted with empty vector (EV), USP18-Myc-Flag, wild-type (WT) or G317S mutant. **(F)** qPCR analysis of transcriptional levels of ISGs in wild-type (WT) and *USP18*-knock out THP - 1 cells complemented with empty vector (EV), USP18- Myc-Flag, wild-type (WT) or G317S mutant, treated with 10 ng/ml IFNα. Data are mean ± SD. *P* values determined by one-way ANOVA. **P < 0.01, ****P < 0.0001. **(G)** Co-immunoprecipitation analysis of interaction between USP18 and ISG15. HEK293T cells were cotransfected with Myc-ISG15 and USP18-Flag, wild-type (WT), G317S and I60N mutant. Immunoprecipitation of ISG15 was performed using Myc antibodies.

The C230X mutation caused the premature termination of USP18 protein, resulting in complete loss of protein function. Therefore, we focused on investigating the pathogenicity of the G317S mutation. To evaluate the impact of G317S mutation on type I IFN signaling, we employed an IFN-stimulated response elements (ISRE)-driven luciferase reporter assay to monitor the transcriptional activity. Upon stimulation with increasing IFNα concentrations, ISRE activity was notably downregulated in cells overexpressing wild-type (WT) USP18 compared to those overexpressing either empty vector (EV) or G317S mutant USP18, illustrating the G317S mutation impaired the suppressing function of USP18 (**Figure 3A**).

Next, we transfected constructs of WT or G317S mutant USP18 into HEK293T cells to assess the level of type I IFN signaling by measuring phosphorylated STAT2 levels via western blotting. Cells expressing G317S mutant USP18 exhibited higher and sustained p-STAT2 levels upon IFNα stimulation across varying concentrations (**Figure 3B**) and time points (**Figure 3C**), compared to those expressing WT USP18 (**Figures 3B, C**). To investigate IFN-induced desensitization, we conducted a priming experiment. Cells overexpressing WT USP18, primed with IFNα, were refractory to second stimulation as evidenced by diminished STAT2 phosphorylation, consistent with desensitization. In contrast, cells overexpressing G317S mutant USP18 exhibited a failure of desensitization of IFNAR2 signaling, with only marginally reduced p-STAT2 levels after restimulation (**Figure 3D**). Additionally, we generated *USP18*-knock out (KO) THP - 1 cell lines and transduced them with lentiviruses encoding either WT or G317S mutant USP18 with Myc-Flag tag in the C terminal. The G317S mutation recapitulated the heightened sensitivity to IFNα, as indicated by elevated STAT1 phosphorylation (**Figure 3E**). Increased expression of ISGs was also confirmed by qPCR assay upon IFNα stimulation in THP - 1 cells reconstituted with G317S mutant USP18 (**Figure 3F**). Reconstitution of *USP18* KO cells with WT USP18 partially restored p-STAT1 levels and ISGs expression to levels observed in WT THP - 1 cells, potentially due to partial interference from the C-terminal Myc-Flag tag affecting USP18 function. Taken together, those results demonstrated G317S mutant USP18 had a failure of negative feedback regulation of type I IFN signaling.

The molecular mechanism underlying the heightened type I IFN sensitivity of the G317S mutant USP18 requires further investigation. Our findings indicated that the G317S mutation did not impair deISGylation enzymatic activity (**Supplementary Figure S1A**) and ISG15-mediated stabilization (**Supplementary Figure S1B**). Co-immunoprecipitation assays demonstrated the G317S mutation maintained interactions with STAT2 and IFNAR2, indicating preserved binding to components of the receptor signaling complex (**Supplementary Figures S1C, D**). Recent studies have revealed that the role of ISG15 as a USP18 stabilizer is insufficient for effective negative regulation of type I IFN signaling, which also requires non-covalent interactions between USP18 and ISG15 C-terminal diGlycine motif (8). Molecular modeling demonstrated that the G317 residue is positioned above the typical catalytic triad of USP18, and substitution of glycine with serine at this site (G317S) alters the polarity of the amino acid (**Figure 1F**), supporting the hypothesis that the G317S mutation might disrupt the USP18:ISG15 interaction. Co-immunoprecipitation assays showed significantly reduced binding between G317S mutant USP18 and ISG15, compared to WT USP18 (**Figure 3G**). To confirm the specificity of the interaction, the I60N mutant of USP18, which is defective in ISG15 binding, was included as a negative control (9). These findings highlighted that the G317S mutation specifically impairs USP18’s regulatory function in type I IFN signaling, primarily due to its altered interaction with ISG15.

### 3.4 Patient was responsive to ruxolitinib

**Figure 4:**
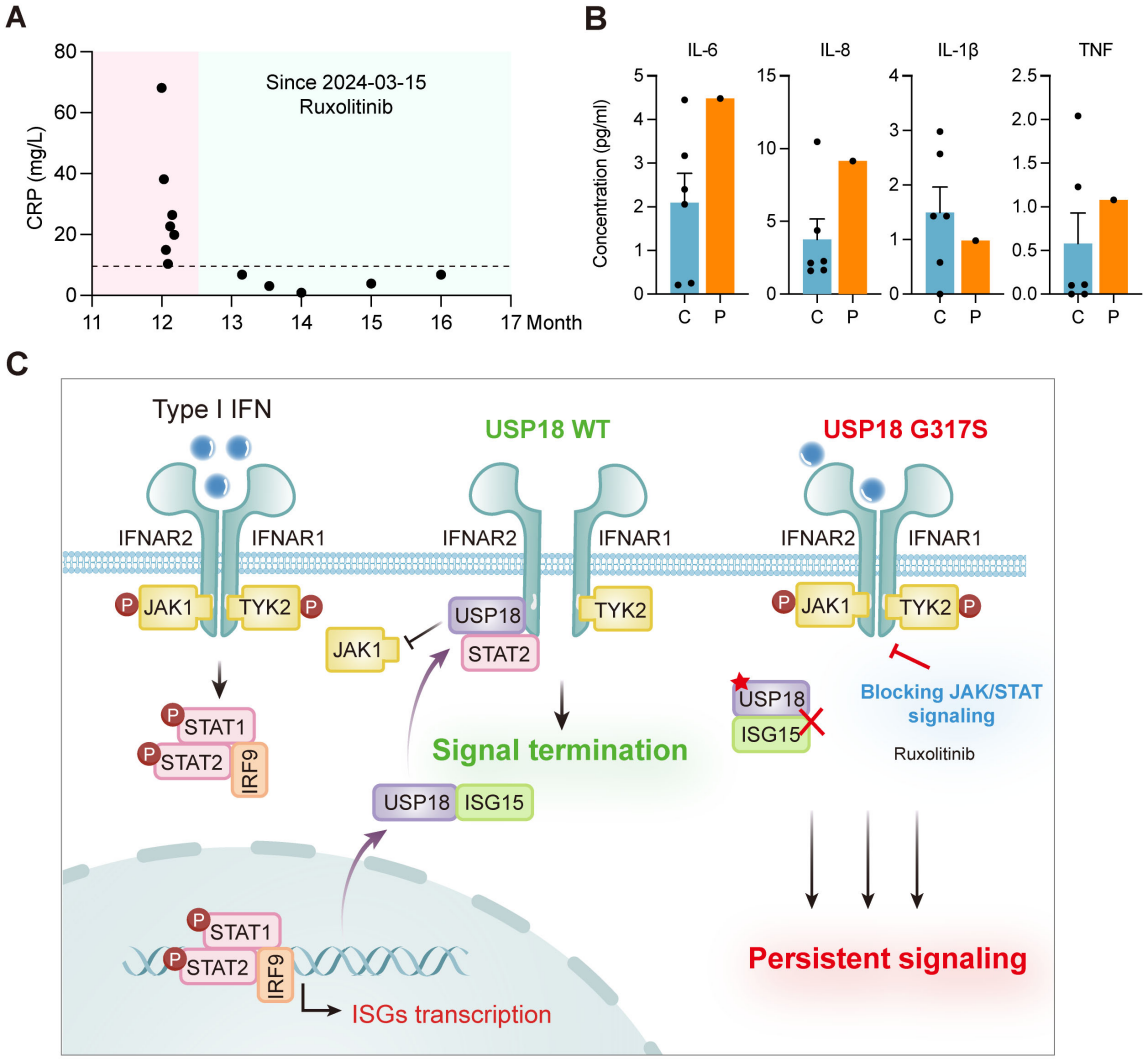
Clinical improvement with ruxolitinib treatment and schematic model of G317S mutant USP18 leading to impaired suppression of type I IFN signaling. **(A)** Levels of C-reactive protein (CRP) of patient P1 (P) before and after ruxolitinib treatment. **(B)** Plasma levels of cytokines (IL - 6, IL - 1β, TNF), and the chemokine IL - 8 of patient P1 (P) following ruxolitinib therapy, as detected by CBA. C: healthy controls. **(C)** Schematic model of USP18 G317S mutation leading to an exaggerated and prolonged response upon type I IFNs exposure and targeted treatment strategy.

Patient P1 developed recurrent respiratory tract infection starting at 6 months of age, with symptoms alleviated by symptomatic treatment. At 12 months, the patient was admitted to the pediatric intensive care unit (PICU) for respiratory failure and multiorgan dysfunction. He received supportive care, antibiotics, and corticosteroids, and was discharged after 21 days of hospitalization. Given the strong type I IFN signature observed in the patient, the identification of biallelic *USP18* mutations and evidence of sustained recovery in a USP18-deficient patient treated with JAK inhibitor ruxolitinib, patient P1 was prescribed oral ruxolitinib (2.5 mg twice daily) alongside symptomatic medications post-discharge. Monthly blood tests at a local hospital monitored his condition. After 7 months of ruxolitinib treatment, the patient showed a marked response, with stable normalization of CRP levels (**Figure 4A**) and reduced inflammatory cytokines and chemokines, including IL - 6, IL - 8, IL - 1β and TNF (**Figure 4B**). After 12 months of ruxolitinib treatment, the laboratory parameters and the clinical manifestations achieved complete remission (**Supplementary Table S1**). The proposed pathogenic mechanism of USP18 G317S mutation and targeted treatment strategy are depicted in a schematic model (**Figure 4C**).

## 4 Discussion

Here, we identified novel biallelic *USP18* mutations in patients with severe early-onset inflammation characteristic of type I interferonopathies. We performed a comprehensive study to confirm the pathogenicity of these mutations and elucidate the underlying molecular mechanism. Treatment with the JAK inhibitor ruxolitinib markedly alleviated the clinical manifestations in patient P1.

Type I IFN signal transduction and ISGs induction are tightly regulated at multiple levels by negative regulators, including ISG15, STAT2 and USP18, which directly participate in the cellular pathway. USP18 deficiency, STAT2 LOF mutations and ISG15 deficiency represent three autosomal recessive disorders that impair negative regulation of type I IFN signaling, leading to a net gain of signaling activity without IFNα/β overproduction (9–11, 16–19). Disease severity correlates strongly with the extent of USP18 dysfunction. USP18 deficiency and STAT2 LOF mutations, which disrupted USP18 interaction, led to extensive dysfunction of USP18 as a negative regulator of IFNAR signaling. Consequently, patients with these mutations manifested with severe neurological and systemic inflammation. In contrast, ISG15 deficiency causes only partial reduction of USP18 protein, resulting in milder disorders such as neurological complications without systemic inflammation (16). In our study, both patients presented with neurological disorders and severe systemic inflammation, with patient P2 succumbing at three months of age. Notably, PBMCs from patient P1 exhibited heightened and prolonged type I IFN signaling activation in response to IFNα stimulation, consistent with a key feature of USP18 deficiency. The phenotypic spectrum in our patients overlapped with previously reported cases of USP18 deficiency (**Supplementary Table S2**). These findings confirmed that the identified biallelic mutations in *USP18* impaired negative regulation and drove type I IFN-dependent autoinflammation.

Recent studies have highlighted the critical role of ISG15 on genome stability, with its dysregulation potentially contributing to pathogenesis (20). High ISG15 levels can induce replication stress, extensive DNA damage, chromosomal aberrations and tumorigenesis (21, 22). ISGylation at replication forks is essential for genomic stability (23). Furthermore, ISG15 deficiency altered the constituents of the replication fork, causing tonic ataxia telangiectasia and Rad3-related (ATR) activation, replication fork stalling, and spontaneous chromosome aberrations (23). Consequently, the elevated levels of conjugated ISG15 observed in patient-derived cells upon IFNα stimulation suggest broader implications. Although DNA damage has not been directly assessed in USP18-deficient cells, the aberrant ISGylation strongly indicates a potential contribution to genomic instability. These findings highlight a plausible mechanistic link between USP18 dysfunction, abnormal ISG15 regulation, and compromised genome integrity, warranting further investigation.

*In vitro* experiments revealed the G317S mutation did not affect catalytical activity, ISG15-dependent stabilization, interactions with STAT2 or IFNAR2, and formation of the IFNAR2/STAT2/USP18 complex. However, G317S mutant USP18 was partially impaired in binding to ISG15. Non-covalent interactions between ISG15 and USP18 have been shown to be essential for negative regulation of type I IFN signaling *in vitro*, although their significance *in vivo* has not yet been demonstrated (8). The I60N mutant USP18 maintained normal expression, ISG15 isopeptidase activity, and ISG15-mediated stabilization, but showed partially impaired STAT2 binding and significantly reduced ISG15 interaction. However, the functional consequences of the disrupted USP18:ISG15 interaction were not addressed (9). Our study proposed a new pathogenic mechanism-reduced USP18:ISG15 binding-which differs from previously reported mechanisms, such as complete absence of USP18 protein, impaired ISG15-dependent stabilization, or diminished STAT2:USP18 interaction. Compared to the C230X mutation on one allele, which results in haploinsufficiency of USP18, the G317S mutation represents a hypomorphic allele. Together, both mutations were pathogenic and resulted in USP18 deficiency.

Currently treatment strategies for type I interferonopathies primarily target the IFN signaling cascade (24). JAK inhibitors, as first-line therapies, effectively reduce ISGs expression by inhibiting JAK1/2-STAT1/2 phosphorylation. The JAK1/2 inhibitor ruxolitinib has proven highly effective in a child with USP18 deficiency by combination with supportive therapy, achieving prolonged survival and resolution of the associated complications, albeit with slower progress in developmental milestones (11). Based on the genetic diagnosis and excessive type I IFN signaling, the affected child in this study began ruxolitinib therapy at 12 months, continuing for over 12 months until now. Introduction of ruxolitinib led to great clinical remission. Early initiation and sustained administration of targeted therapy appeared to prevent neurological sequelae and avoid irreparable damage. Ongoing monitoring of molecular markers and regular clinical follow-up are crucial to evaluate long-term therapeutic response.

In summary, our study identifies novel USP18 mutations in two siblings with early-onset systemic inflammation and uncovers a previously unrecognized pathogenic mechanism. The efficacy of ruxolitinib in alleviating symptoms highlights its potential as a therapeutic option for patients with USP18 deficiency and other type I interferonopathies.

## Data Availability

The raw RNA-seq data presented in the study are deposited in the Genome Sequence Archive (25) in National Genomics Data Center (26), China National Center for Bioinformation / Beijing Institute of Genomics, Chinese Academy of Sciences, accession number GSA-Human: HRA012983. All other relevant data are available in the main text or the supplementary materials.
