# Supplementary material for: Novel USP18 mutations lead to severe interferonopathy responsive to JAK inhibitor
Source: Front Immunol. 2025 Sep 4;16:1646996. doi: 10.3389/fimmu.2025.1646996 (PMC12443803; doi:10.3389/fimmu.2025.1646996)
Supplement: Supplementary file 1 [file DataSheet1.pdf]

Supplemental materials

Table S1: Laboratory features of patient P1.

Laboratory features	Pre ruxolitinib	Post ruxolitinib	Reference
**Hematology**			
White blood cell count (WBC)	4.18↓	6.01	5.5-13.6 (10^9/L)
Red blood cell count (RBC)	2.95↓	4.12	4.1-5.5 (10^12/L)
Hemoglobin (HGB)	82↓	106↓	104-143 (g/L)
Hematokrit (HCT)	23.2↓	31.0↓	32-43 (%)
Platelet count (PLT)	23↓↓	356	191-516 (10^9/L)
Lymphocytes (%)	14.1↓	75.0	35-76 (%)
Neutrophil (%)	80.2↑	16.0	13-54 (%)
Monocytes (%)	5.7	7.0	2–14 (%)
Eosinophil (%)	0↓	1.0	0.5–9 (%)
Basophils (%)	0	0	0.00–1.00 (%)
Reticulocyte count (RET)	0.4↓	NA	0.5-1.5 (%)
**Biochemistry**			
Activated partial thromboplastin time (APTT)	70.7↑↑	NA	22.3-32.5 (s)
Fibrinogen	1.62↓	NA	2-4 (g/L)
D-dimer	4.14↑	NA	0-0.55 (mg/LFEU)
C-reactive protein (CRP)	36.68↑	<0.77	<8 (mg/L)
Erythrocyte Sedimentation Rate (ESR)	49↑	NA	0-15 mm/60min
Ferritin (SF)	7438.19↑	40.80	10-120 ng/ml
Creatinine (CREA)	42.8↑	24.8	13-33 (μmol/L)
Alanine aminotransferase (ALT)	91.8↑	20.0	8-42 (U/L)
Aspartate aminotransferase (AST)	268.9↑	39.5	22-59 (U/L)
Albumin (ALB)	28.8↓	48.6	39-54 (g/L)
Globulin (GLB)	14.7	24.8	N/A (g/L)
Total protein (TP)	43.5↓	73.4	58-76 (g/L)
Lactate dehydrogenase (LD)	1230↑	318	80-300 (U/L)
Uric Acid (UA)	389↑	239	155-344 (μmol/L)
**Inflammatory markers**			
**Serum**			
TNF-α	111↑	<2.44	0-8.1 pg/ml
IL-6	>1000↑	<2.44	0-3.4 pg/ml
IL-2R	7209↑	NA	223-710 U/ml
IL-8	921↑	<2.44	223-710 pg/ml
IL-10	76.2↑	<2.44	0-9.1 pg/ml
IL-1β	60.3↑	<2.44	<5 pg/ml
**Cerebrospinal Fluid (CSF)**			
TNF-α	5.15	NA	<16.5 pg/ml
IL-6	75.73↑	NA	<5.4 pg/ml
IL-2	<2.44	NA	<7.5 pg/ml
IL-8	12.9	NA	<20.6 pg/ml
IL-10	<2.44	NA	<12.9 pg/ml
IL-1β	24↑	NA	<12.4 pg/ml
IFN-α	<2.44	NA	<8.5 pg/ml
IFN-γ	<2.44	NA	<23.1 pg/ml
**Microbiology**			
Staphylococcus aureus	Positive	NA	

Laboratory features	Pre ruxolitinib	Post ruxolitinib	Reference
Influenza B virus (FluB)	Positive	NA	
**Immunology**			
C3 (g/L)	0.88↓	1.330	0.9-1.8 (g/L)
C4 (g/L)	0.23	0.270	0.1-0.4 (g/L)
Immunoglobulin G (IgG)	12.600	8.8700	4.82-12 (g/L)
Immunoglobulin M (IgM)	0.449	0.480 ↓	0.54-2.09 (g/L)
Immunoglobulin A (IgA)	0.730	0.470	0.22-1.18 (g/L)
#CD3+ cells	619.83↓	2934.50	1794-4247 (/uL)
#CD3+ CD4+ cells	381.06↓	1771.81	902-2253 (/uL)
#CD3+ CD8+ cells	226.18↓	934.43	580-1735 (/uL)
#CD3- CD19+ cells	101.10↓	1020.11	461-1456 (/uL)
#CD3- CD56+ cells	31.04↓	208.84↓	270-1053 (/uL)
#CD45	755.97↓	4216.33	2790-6350 (/uL)
%CD3+ cells	82↑	70↑	56.0-68.0 (%)
%CD3+ CD4+ cells	50↑	42↑	29-40 (%)
%CD3+ CD8+ cells	30↑	22	19-25 (%)
%CD3- CD19+ cells	13.37↓	24.19	18.5-28 (%)
%CD3- CD56+ cells	4↓	5↓	9.0-19.0 (%)
#Treg	4.80↓	6.60	5-10 (/uL)
**Autoimmune autoantibodies**			
Rheumatoid factor (RF)	<8.69	<8.56	<15.9 (IU/ml)
Antinuclear Antibody (ANA)	1:100 (-)	NA	Negative <1:100
Anti-Smith Antibody (Sm)	Negative (-)	NA	Negative
Anti-nuclear Ribonucleoprotein Antibody (nRNP)	Negative (-)	NA	Negative
Anti-Ro Antibody (SSA)	Negative (-)	NA	Negative
Anti-La Antibody (SSB)	Negative (-)	NA	Negative
Anti-ScL-70 Antibody	Negative (-)	NA	Negative
Anti-Jo-1 Antibody	Negative (-)	NA	Negative
Anti-double-stranded DNA Antibody (dsDNA)	Negative (-)	NA	<100 (IU/mL)
Anti-Centromere Protein B Antibody (CENP B)	Negative (-)	NA	Negative
Anti-Histone Antibody	Negative (-)	NA	Negative
Anti-Pib.P-pro Antibody	Negative (-)	NA	Negative
Proliferating Cell Nuclear Antigen (PCNA)	Negative (-)	NA	Negative
Anti-Mitochondrial Antibody M2 (AMA-M2)	±	NA	Negative
Anti-Ro-52 Antibody	±	NA	Negative
Anti-PM/SCL Antibody	Negative (-)	NA	Negative
Anti-Nukleosomen Antibody	Negative (-)	NA	Negative

Abbreviations: NA, not available.

Table S2: Clinical and genetic characteristics of patients with *USP18* loss-of-function mutations.

	**Family 1**			**Family 2**		**Family 3**	**Family 4**			**Family 5**	
Described from	*J Exp Med*, 2016(1)					*N Engl J Med*, 2020(2)	*J Exp Med*, 2022(3)			this study	
Mutation	p.Q218X/p.Q218X			p.Q218X/cryptic 3′ del		ΔEx10	p.I60N/p.I60N			p.G317S/p.C230X	
Geographic origin	Turkey			Germany		Saudi Arabia	Morocco			China	
Age onset	Antenatal	Day 1	Day 1	Antenatal	Antenatal	Day 13	1 M			6 M	3 M
Gender	Female	Male	Male	Male	Male	Male	Male	Male	Female	Male	Male
Age at death	23 5/7 GW	1 wk	17 d	22 d	12 d	Live	Live (23 yr)	4 M	9 yr	Live (2 yr)	3 M
Diagnosis	pseudo-TORCH syndrome					Type I interferonopathies	Type I interferonopathies, MSMD			Type I interferonopathies	
Inflammation	Central nervous system inflammation	Systemic inflammation	Systemic inflammation	Systemic inflammation	Systemic inflammation	Central nervous system inflammation	Central nervous system inflammation	Systemic inflammation	Systemic inflammati on	Systemic inflammation	NA
Infection	NA	NA	NA	NA	NA	Bordetella pertussis positive, Enterobacter cloacae, Pseudomonas aeruginosa, Haemophilus parainfluenzae, and Candida parapsilosis	BCG vaccination	BCG vaccination, staphylococcal infection	BCG vaccination, respiratory infection	Staphylococcus aureus and Influenza B virus	NA
Hematologic/c hemistry abnormalities	NA	Thrombocytopenia, AST/ALT/ammonia and lactate elevation	AST/ALT/ammonia and lactate elevation	Thrombocytopenia, dyserythropoiesis on bone marrow aspiration	Thrombocytopenia	Disseminated intravascular coagulopathy, ESR:27 (0-15 mm/h), CRP:13.2 (<10 mg/L)	NA	NA	NA	Leukopenia, thrombocytopenia, erythrocytopenia, capillary leak syndrome, elevated D-dimers, hetabolic acidosis, hypocalcemia, hypophosphatemia, hyponatremia, hypokalemia	Disseminated intravascular coagulopathy
Neurological disorder	Microcephaly, SC, BG, and PV calcification, cortical destruction	Seizures, BS, BG, CBL, IV, P hemorrhage, no clear calcifications, normal gyration	Seizures, P, CBL, BG hemorrhage, no clear calcifications, cortical necrosis, PV white matter abnormalities, loss of white/gray matter demarcation	Seizures, massive cerebral hemorrhage, enlarged lateral ventricles, cerebellar hypoplasia, malformations BS and PF, calcifications	Seizures, periventricular signal intensities due to multiple small hemorrhages, septum pellucidum cyst, cavum vergae, lack of gyration of temporal lobes, diminished gyration of parietal lobes	Septic shock，grade II intraventricular hemorrhage, seizures, acute hydrocephalus, mixed hemorrhagic and ischemic damage	BG intracranial calcifications	NA	Severe learning and motor disabilities	CBL, BG intracranial calcification, enlarged ventricles	Cerebral edema, seizures
Respiratory system disorder	NA	Respiratory distress (no spontaneous respiration)	Respiratory distress (ventilation within hours)	Respiratory distress (ventilation within hours)	Respiratory distress (no spontaneous respiration)	Acute respiratory distress syndrome (ARDS)	NA	NA	NA	Pneumonia, pleural effusion, respiratory failure requiring ventilation	Pneumonia, respiratory failure requiring ventilation
Renal disorder	NA	NA	NA	NA	NA	NA	NA	NA	NA	Acute renal insufficiency	NA
Cardiological disorder	NP	PDA, bradycardia	ASD type II, PDA, bradycardia, widening of right and left coronary artery	NP	NP	NA	NA	NA	NA	Acute heart failure	NA
Gastrointestin al disorder	NP	Liver dysfunction, ascites	Liver dysfunction, ascites	Hepatomegaly, ascites	Hepatomegaly, ascites	NA	NA	NA	NA	Splenomegaly, hepatomegaly, liver dysfunction, hypoalbuminemia, gastrointestinal dysfunction	Hypoalbuminemia

	**Family 1**			**Family 2**		**Family 3**	**Family 4**			**Family 5**	
Interferon signature	Elevated	Elevated	NA	NA	NA	Elevated	Elevated	NA	NA	Elevated	NA
Therapy	NA	NA	NA	NA	NA	Ruxolitinib, 10 mg	No	NA	NA	Ruxolitinib, 5 mg	NA
Prognosis	Dead	Dead	Dead	Dead	Dead	Full remission	Full remission	Dead	Dead	Remission	Dead

Abbreviations: NA, not available; NP, not performed; BG, basal ganglia; BS, brain stem; CBL, cerebellum; GW, gestational week; IV, intraventricular; P, parenchymal; PDA, persistent ductus arteriosus; PF, posterior fossa; PV, periventricular; SC, subcortical; ASD, atrium septum defect; ALT, alanine aminotransferase; AST, aspartate aminotransferase; CRP, C-reactive protein; ESR, Erythrocyte Sedimentation Rate

Figure S1

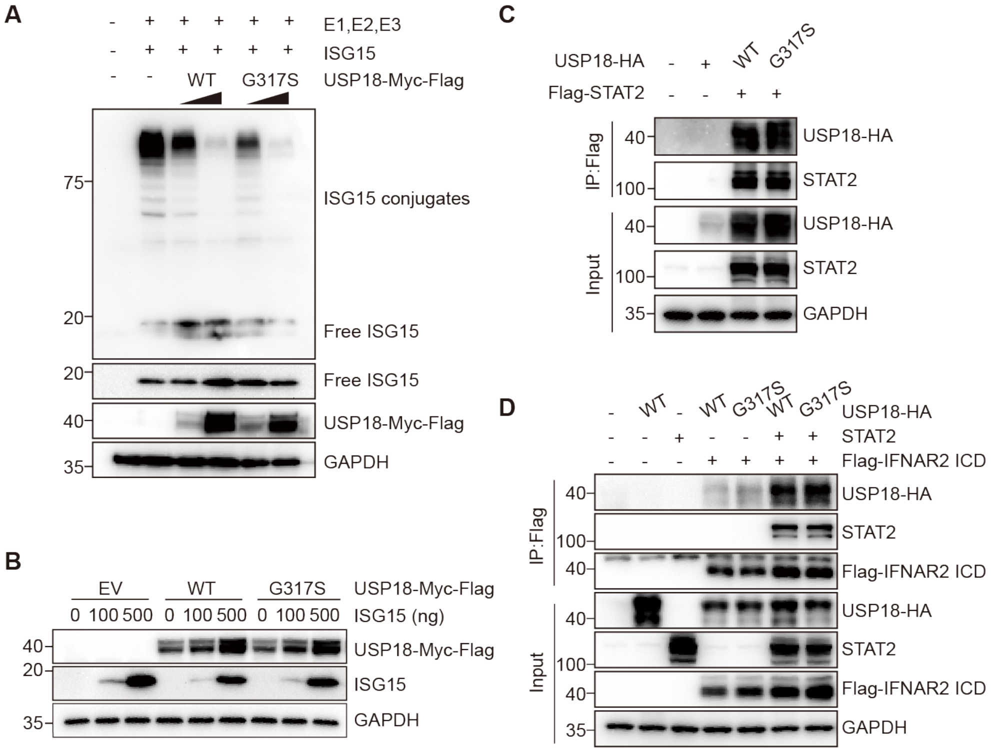

Fig S1. Characterization of the enzymatic and regulatory function of USP18.

(A) Western blotting of ISG15 conjugates in HEK293T cells transfected with ISGylation system (UbE1L (E1), UbCH8 (E2), HerC5 (E3), and ISG15) and either WT or G317S mutant USP18. **(B)** USP18 expression level in HEK293T cells transfected with increasing amounts of ISG15 analyzed by Western blotting. **(C)** Co-immunoprecipitation analysis of interaction between USP18 and STAT2. HEK293T cells were cotransfected with Flag-STAT2 and USP18-HA, WT or G317S. STAT2 was immunoprecipitated using Flag antibodies. **(D)** Co-immunoprecipitation analysis of interaction between USP18 and IFNAR2 and the formation of the IFNAR2/STAT2/USP18 complex. HEK293T cells were cotransfected with USP18-HA, WT or G317S, Flag-IFNAR2 intracellular domain (ICD), together with or without STAT2. IFNAR2 was immunoprecipitated using Flag antibodies.

Alt text: Immunoblotting was used to assess USP18 enzymatic activity and ISG15-mediated stabilization. Co-immunoprecipitation was performed to examine the

formation of complex USP18/STAT2/IFNAR2 and the interaction between USP18 and STAT2 or IFNAR2.

Reference

1. Meuwissen MEC, Schot R, Buta S, Oudesluijs G, Tinschert S, Speer SD, Li Z, van Unen L, Heijsman D, Goldmann T, et al. Human USP18 deficiency underlies type 1 interferonopathy leading to severe pseudo-TORCH syndrome. *J Exp Med* (2016) 213:1163–1174. doi: 10.1084/jem.20151529

2. Alsohime F, Martin-Fernandez M, Temsah M-H, Alabdulhafid M, Voyer TL, Alghamdi M, Qiu X, Alotaibi N, Alkahtani A, Buta S, et al. JAK Inhibitor Therapy in a Child with Inherited USP18 Deficiency. *New England Journal of Medicine* (2020) 382:256–265. doi: 10.1056/NEJMoa1905633

3. Martin-Fernandez M, Buta S, Le Voyer T, Li Z, Dynesen LT, Vuillier F, Franklin L, Ailal F, Muglia Amancio A, Malle L, et al. A partial form of inherited human USP18 deficiency underlies infection and inflammation. *J Exp Med* (2022) 219:e20211273. doi: 10.1084/jem.20211273
